# Periviable delivery of a pregnancy in a rudimentary uterine horn: A case report

**DOI:** 10.1016/j.crwh.2021.e00346

**Published:** 2021-07-24

**Authors:** Katherine E. Coakley, Tyler B. Yang, Judith H. Chung

**Affiliations:** University of California, Irvine Medical Center, Department of Obstetrics and Gynecology, Division of Maternal Fetal Medicine, 333 City Boulevard West, Suite 1400, Orange, CA 92868, United States of America

**Keywords:** Case report, Rudimentary uterine horn pregnancy, Periviability

## Abstract

Pregnancy in a rudimentary uterine horn is an extremely rare form of ectopic pregnancy, with an incidence of 1 in 76,000–140,000 pregnancies. Given its high-risk nature, the standard of care is to terminate such pregnancies at the time of diagnosis. This is a case of a nulliparous patient at 23 5/7 weeks of gestation with a new diagnosis of a rudimentary horn pregnancy. She elected to proceed with full intervention for her fetus and was delivered at 24 0/7 weeks after administration of antenatal corticosteroid therapy. While the infant did have some adverse effects related to prematurity, she met developmental milestones and was alive and well at the age of two. Although the standard of care is to manage these cases as ectopic pregnancies, when diagnosed at a periviable gestational age, optimization of fetal status prior to delivery may be an alternative approach to immediate delivery.

## Highlights

• Pregnancy in a rudimentary uterine horn is a rare type of ectopic pregnancy with poor maternal and neonatal outcomes.
• The standard recommendation is for pregnancy termination with removal of the uterine horn at the time of diagnosis.
• When diagnosed at a periviable gestational age, optimization of fetal status and cesarean delivery may be considered.
• Abnormal placentation is common, and thus the delivering physician should be prepared for this possible complication.

## 1 Introduction

A class II Mullerian anomaly occurs when one side of the Mullerian tract fails to develop [1]. As a result, there is a unicornuate uterus and a rudimentary uterine horn [1]. The rudimentary horn can be further classified as to whether or not it contains an endometrial cavity and, if present, whether the cavity of the rudimentary horn communicates with the main cavity of the unicornuate uterus. Where the rudimentary horn has a cavity, more often than not there is no direct communication to the main cavity of the unicornuate uterus [1]. Pregnancy in a rudimentary uterine horn is estimated to occur in only 1 in 76,000 to 1 in 140,000 pregnancies and is believed to result from transperitoneal migration of sperm from the contralateral fallopian tube [2]. The prognosis for such pregnancies is poor, with the most common outcome being uterine rupture [3]. This occurs in over 50% of such pregnancies, with up to 80% of these ruptures occurring in the second trimester [3]. As such, the estimated neonatal survival is only 6% [3]. Given the high-risk nature of these pregnancies, they are traditionally managed as ectopic pregnancies, and surgical removal of the rudimentary horn with the pregnancy in situ is performed at the time of diagnosis.

## 2 Case Presentation

**Fig. 1:**
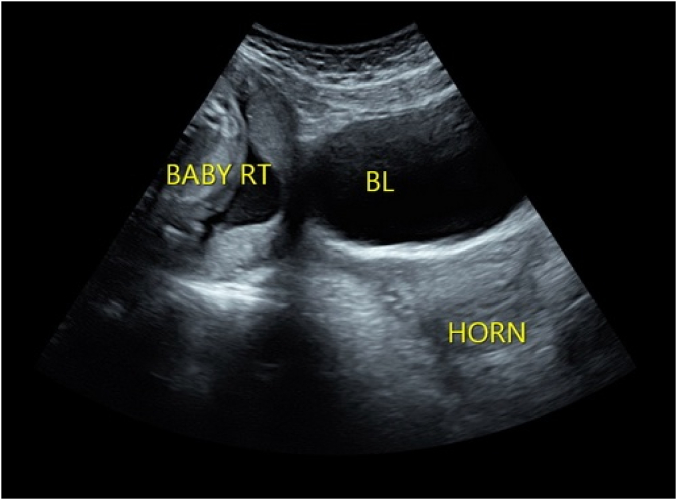
Ultrasound image from the dates and anatomy scan at 23 3/7 weeks of gestation with sonographer annotations. Baby RT demonstrates the pregnancy in the uterine horn on the maternal right, BL is the maternal bladder, and horn is the empty uterus.

A 26-year-old woman, G1P0, at 23 4/7 weeks of gestation presented to Labor and Delivery as a maternal transport for a suspected abdominal pregnancy. Her past history was significant for the patient's report of surgery at 3 years of age to “separate [her] vagina from [her] rectum.” At an outside facility, she had undergone a dates and anatomy ultrasound scan at 20 3/7 weeks of gestation and was scheduled for a follow-up at 23 3/7 weeks for completion of the anatomic survey. At this follow-up ultrasound examination, she was thought to have a bicornuate uterus with a suspected abdominal pregnancy. Fig. 1 demonstrates a pregnancy to the left of an empty uterus, labeled “horn.” During the examination, she was noted to have pain and was therefore sent to the Emergency Department for further evaluation. Magnetic resonance imaging (MRI) showed “a well circumscribed extra-uterine gestation with abnormal appearing placentation and no definite communication with the endometrial cavity, most consistent with abdominal ectopic pregnancy.” Due to these findings, the patient was transferred to a higher-level hospital.

**Fig. 2:**
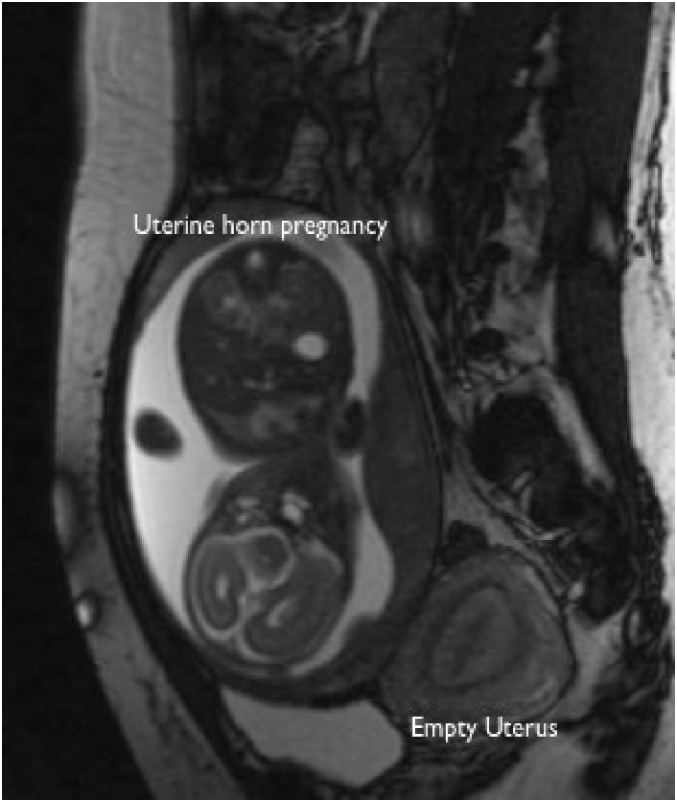
Magnetic resonance scan showing empty uterus with pregnancy located anterior to the uterus.

Upon arrival, a bedside ultrasound scan performed by Maternal-Fetal Medicine showed a left-sided empty uterus with endometrial lining connecting to the cervix. A pregnancy was visualized to the right of the uterus, without a visible connection between the pregnancy and the empty uterus. The pregnancy was mobile, independent of the left uterus as well as the surrounding bowel. A thin rim of tissue was noted to be surrounding the pregnancy, and no placental vessels could be seen invading adjacent, intra-abdominal structures. Therefore, abdominal pregnancy was thought to be less likely. Repeat MRI showed findings suggestive of a pregnancy within a rudimentary right horn of a unicornuate uterus. Fig. 2 is a representative image from this study showing a small empty uterus and a singleton gestation, thought to be within a rudimentary uterine horn.

The patient was counseled by Maternal-Fetal Medicine and Neonatology. Pregnancy options were discussed, including pregnancy termination, which she declined. Instead, the patient desired optimization of fetal status, understanding the potential risk of catastrophic uterine rupture with continuation of pregnancy. Given that the patient did not have abdominal tenderness, antenatal corticosteroid therapy was administered at 23 5/7 and 23 6/7 weeks of gestation. After a meeting of the Ethics Committee, the decision was made to proceed with delivery at 24 0/7 weeks.

**Fig. 3:**
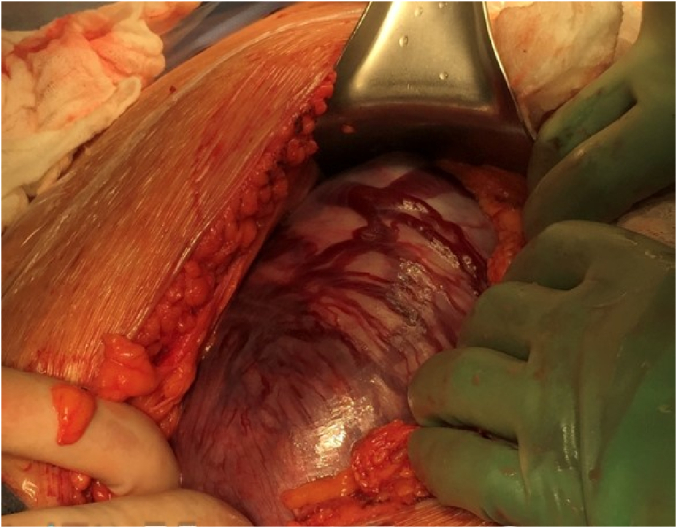
Findings upon exploratory laparotomy: the uterine horn containing the pregnancy with prominent vasculature on the serosal surface.

**Fig. 4:**
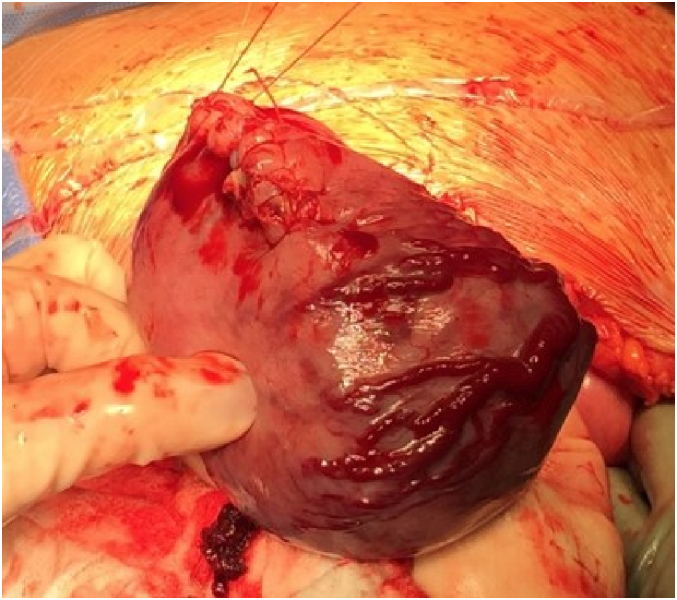
The uterine horn following delivery, closed via running whipstitch.

**Fig. 5:**
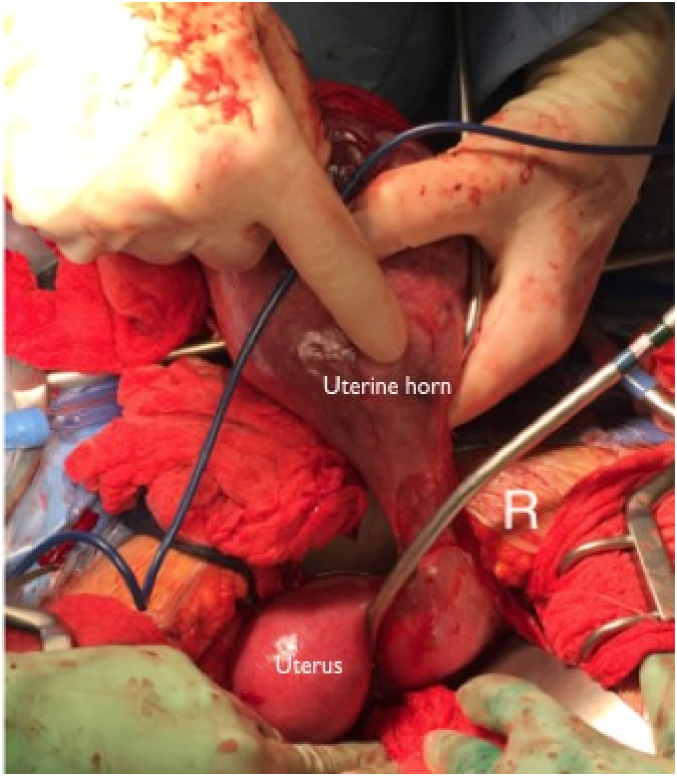
Uterine horn originating off the right side of the unicornuate uterus.

The patient underwent an exploratory laparotomy via vertical midline skin incision. A left unicornuate uterus with a pregnancy in the right rudimentary horn was noted. A thin stalk from the rudimentary horn to the unicornuate uterus was noted measuring 2.5 cm in length and 1 cm in width. Fig. 3 shows the intact uterine horn upon opening the abdominal cavity. Prominent vasculature was noted on the serosal surface of the horn. Vertical hysterotomy of the rudimentary horn was performed, taking care to avoid the areas of prominent vasculature. The myometrium was noted to be very thin upon entry. A viable female infant was then delivered with Apgar scores of 1 at one minute, 5 at 5 min, 6 at 10 min, and 7 at 20 min; birthweight was 534 g. Delayed cord clamping was not performed due to suspected abnormal placentation at the time of delivery. The placenta was left in situ, the hysterotomy was closed (Fig. 4), and the rudimentary uterine horn was excised at its insertion site into the right side of the unicornuate uterus (Fig. 5).

Final pathology revealed firmly adherent placenta to the endometrium of the excised uterine horn, consistent with placenta accreta. The infant's postnatal course was complicated by respiratory distress syndrome, patent foramen ovale, necrotizing enterocolitis stage 1, and retinopathy of prematurity. Head ultrasound was negative for intraventricular hemorrhage. Ultimately, the infant was discharged on day of life 112 (40 0/7 weeks).

## 3 Discussion

A unicornuate uterus, or class II Mullerian anomaly, results from failed development of one side of the Mullerian ducts [1]. Its prevalence is approximately 1/250–1/4020 women [4]. In 75%–92% of cases, the rudimentary uterine horn has an endometrial cavity that does not communicate with the cavity of the unicornuate uterus [5]. In the remaining cases, the rudimentary horn may not have an endometrial lining, or it may have a lining that communicates directly to the endometrial cavity of the unicornuate uterus. In rare cases, the unicornuate uterus may not be accompanied by an adjacent uterine horn [5].

Diagnosis is typically made in the third decade of life and is most commonly discovered after development of symptoms or in the setting of an abnormal pregnancy [5]. According to one review, diagnosis prior to development of symptoms occurs in only 14% of cases [5]. The standard imaging modality for diagnosis of a rudimentary uterine horn is ultrasound. Tsafrir et al. described three criteria for ultrasound diagnosis of a pregnancy in a rudimentary uterine horn: pseudo-pattern of an asymmetrical bicornuate uterus, absent visual continuity between the cervical canal and the lumen of the pregnant horn, and the presence of myometrial tissue surrounding the gestational sac, all of which were present in our case [6]. However, the sensitivity of ultrasound for the detection of a rudimentary horn is reported to be only 26% [5]. Thus, supplemental MRI has also been suggested for improved diagnostic accuracy [6].

Ideally, the diagnosis of a rudimentary horn would be made prior to conception, and in such cases resection is recommended. This is most often accomplished laparoscopically [6]. This patient had reported a history of surgery at 3 years of age to “separate [her] vagina from [her] rectum.” Records from this surgery were obtained and reviewed, revealing that she had undergone a repair of a cloacal abnormality by Urology and Colorectal Surgery. It did not appear that any abnormalities of the patient's uterus were identified at that time. Given the high concomitant incidence of Mullerian anomalies with abnormalities of the vagina and urinary tract [7], patients undergoing such corrective surgeries may benefit from preoperative gynecologic evaluation.

Pregnancy in a rudimentary uterine horn is extremely rare and has historically poor outcomes. With early diagnosis, the standard of care is termination of the pregnancy. Most case reports have described laparoscopy with intact surgical resection of the uterine horn as the treatment of choice, although there is one report of a medical abortion with mifepristone and misoprostol, followed by delayed surgical excision of the horn [1]. For those pregnancies that continue beyond the first trimester, the rupture rate approaches 80%, leading to severe maternal and perinatal morbidity and mortality [3]. Those that are diagnosed at viability are often incidentally found or present with uterine rupture [3]. Furthermore, the high incidence of abnormal placentation in uterine horn pregnancies, as occurred in this case, may require additional consideration [8].

Expectant management of a known pregnancy in a rudimentary horn is not generally practiced. This case presented a unique challenge in that the diagnosis was made at a periviable gestational age. Although immediate pregnancy termination was recommended, the patient strongly desired optimization of fetal status. Given the complicated discussion regarding maternal risk of continuation of pregnancy and neonatal risk of iatrogenic preterm delivery, we chose to involve the institutional Ethics Committee in our discussions. This was an important step that allowed for a multidisciplinary approach to decision-making in conjunction with Maternal-Fetal Medicine, Neonatology, and the patient. Weighing the risk of uterine rupture to both mother and fetus, with the likelihood of fetal death and severe maternal morbidity if rupture were to occur, the recommendation was made for delivery under controlled circumstances at 24 0/7 weeks, after the administration of antenatal corticosteroids. The intervention was successful, and the infant was well two years following delivery. She had bronchopulmonary dysplasia and retinopathy of prematurity but was otherwise meeting normal developmental milestones. Thus, for uterine horn pregnancies diagnosed in the periviable period, administration of antenatal corticosteroids followed by controlled delivery may be an option for women strongly desiring optimization of fetal status. However, maternal stability is a prerequisite, maternal understanding of the potential risks is imperative, and immediate availability of Maternal-Fetal Medicine, Anesthesia, Neonatology, and Blood Bank services is necessary.
